# Network-based identification of hub transcription factors associated with benzylisoquinoline alkaloid biosynthesis in *Papaver somniferum*

**DOI:** 10.1016/j.bbrep.2025.102147

**Published:** 2025-07-23

**Authors:** Mahsa Eshaghi, Sajad Rashidi-Monfared

**Affiliations:** Department of Plant Biotechnology, Faculty of Agriculture, Tarbiat Modares University, Tehran, Iran

**Keywords:** Benzylisoquinoline alkaloids, Co-expression and Co-Regulation networks, Hub TFs, Opium poppy

## Abstract

*Papaver somniferum* L. (Opium poppy) is one of the world's most economically valuable medicinal plants, and it is the industrial source of essential compounds such as painkillers and other pharmecutical drugs. Recently, large amounts of opium poppy transcriptome data have become accessible in public databases, consisting of data on the different tissues and ecotypes. Despite the importance of this plant, there is little information about the regulatory mechanisms involved in secondary metabolites, especially in benzylisoquinoline alkaloids (BIAs) biosynthesis at the omics level in opium poppy. Herein, we employed co-expression and co-regulation network analysis using Weighted Gene Co-expression Network Analysis (WGCNA) to infer and reveal gene interactions in opium poppy by using RNA-Seq data. To validate possible hub transcription factors (TFs), partial least squares regression (PLS) and Receiver operating characteristic (ROC) analyses were conducted. We identified nine significant co-regulated modules (comprising1501 genes) and hub TF genes related to the biosynthesis of BIAs, including WRKY3, WRKY32, MYB3R-5, bZIP, APRR2, MYB43, MYB82, bHLH, and WRKY40. The results suggest that these hub genes can play a vital role in co-regulating genes involved in secondary metabolic pathways in opium poppy. Also, we detected common regulatory motifs related to hub TFs (WRKY and MYB) of important co-regulated BIA modules. We implied their common regulatory role in the biosynthesis of secondary metabolites in the opium poppy. The results illustrated that co-expressed genes of the modules share common regulatory motifs, especially related to hub TFs of each module, and that they may define their common regulation. ROC analysis with high diagnostic value (AUC = 1) identified the possible role of the hub TF involved in the BIAs pathways. PLS analysis showed a considerable effect of hub TFs (WRKY and bZIP) on the related genes. Our WGCNA analysis at the omics level, along with identifying hub TFs, highlights the regulatory potential of these genes and the primary molecular mechanisms involved in the BIA biosynthetic pathway in opium poppy. These findings provide valuable insights for the regulon engineering of candidate hub TFs in expression systems.

## Highlights

• We have conducted co-expression network analysis in different transcriptome data of *Papaver somniferum* L.
• WGCNA analysis revealed WRKY and MYB hub TFs regulate nine modules in Benzylisoquinoline alkaloids biosynthesis.
• Consensus motifs related to Benzylisoquinoline alkaloid modules were identified.
• This result could be applied in metabolic engineering to improve secondary metabolite.

## Graphical abstract

**Figure:**
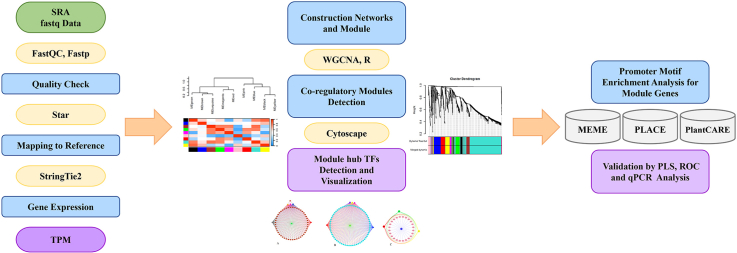

## 1 Introduction

The *Papaver somniferum* L. (opium poppy), a member of the Papaveraceae family, is a conspicuous medicinal plant and a major source of pain-relieving compounds globally [1]. Approximately 40 types of alkaloid compounds have been identified in poppy, including morphine, papaverine, noscapine, protoberberines, protopines, sanguinarine, etc [2]. These biologically active compounds have been used in pain management, cough suppression, and the treatment of conditions like diarrhea and cancer [[3], [4], [5]]. The opium poppy exhibits pharmacological properties such as antioxidant, antimicrobial, anti-inflammatory, anticancer, and neuroprotective effects, primarialy due to its pharmacologically active alkaloids and phenolic compounds [[2], [3], [4], [5], [6]]. Benzylisoquinoline alkaloids (BIAs) based on the nitrogen group are present in eudicots, especially in the Ranunculales order (Ranunculaceae, Papaveraceae, Menispermaceae, and Berberidaceae families). However, BIAs are found in the eumagnoliids order and some Piperales order [6]. BIAs pathway includes a variety of specific restricted enzymes that catalyze pair reactions and functional group modification. BIA starts with the decarboxylation of tyrosine or DOPA to produce dopamine, which, together with 4-hydroxyphenyl acetaldehyde, an aldehyde obtained from tyrosine, is changed to reticuline. Further conversion in the different alkaloids is carried out by some complex enzyme activities, including A-methyl, N-methyl, and A-acetyltransferase, cytochrome p450, FAD-dependent oxidases, non-heme oxygenases, NADPH-dependent reductases [2,[6], [7], [8]]. The biosynthesis of secondary metabolites in medicinal plants has been examined in several recent studies [[9], [10], [11], [12]].

High-throughput expression data technology, such as next-generation sequencing (NGS) allows us to understand the gene's dynamic behavior [13]. Today, systems biology has been used to figure out the biological mechanisms in a cellular system [14]. The co-expression network explains the relationship among genes. One of the important aims of expression network analysis is to discover how key genes regulate or affect the expression of other genes [15]. The correlation-based weighted gene co-expression network is one of the advanced statistical analyses to identify similar patterns among genes. Graph analysis techniques are then utilized to identify strong connections (modules) among genes in co-expression networks, and investigative hub nodes with significant levels of connectivity within the network [16]. The WGCNA method can overcome the complexity of a dataset from hundreds of genes to fewer modules [17]. The weighted network approach has been applied to recognize modules of the gene in various medicinal plants, inducing *Camellia sinensis*, *Narcissus tazetta*, *Salvia castanea*, *Chrysanthemum × morifolium, Angelica dahurica*, *Litsea coreana*, *Valeriana officinalis***,** and *Crocus sativus,* which belong to the secondary biosynthetic pathways [[18], [19], [20], [21], [22], [23], [24], [25], [26]]. In maize and saffron, this approach has been used to identify new genes and regulatory mechanisms of inositol phosphate metabolism and the flowering process [26,27].

There are some different studies on opium poppy, however, there is not much information about regulatory mechanisms in poppy. Also, no comprehensive study has been conducted on the co-regulatory transcriptional network of opium poppy in stem and capsule tissues, which are the primary producers of major metabolites, until now. The present study is the first research in this field, based on this plant being native to regions of Iran and Afghanistan, exhibiting the greatest plant diversity within this geographical region. So, this plant's metabolic engineering of secondary metabolites is necessary for research in this field. Lately, much transcriptome data has been available on opium poppy to construct co-expression genes and co-regulatory networks related to secondary metabolites in public databases. In the present study, we gathered 72 transcriptome data sets on *P. somniferum* in the stem and fruit tissues from the NCBI SRA database (Table S1). Also, we applied in silico methods to establish co-expression and co-regulatory networks and identified modules and highly connected hub genes related to secondary metabolic pathways in opium poppy. Furthermore, we used online tools to discover special *cis*-elements in each module and explore the crucial co-regulatory mechanism of secondary metabolites in opium poppy. Thus, this method can provide novel insights into recognizing effective interactions between hub genes and their target in complex co-regulatory networks in opium poppy. The outcomes of this study are a key preliminary step toward metabolic engineering that involves identifying the transcription factors and main regulators of the complex biosynthetic pathways of benzylisoquinoline alkaloids (BIAs) in this plant.

## 2 Materials and methods

### 2.1 Data collection

Transcriptomic sequence data of *P. somniferum* were downloaded from the National Center for Biotechnology Information (NCBI) Sequence Read Archive database. The data were generated using the Illumina platform with a paired-end read library layout (details are provided in Table S1). NGS Raw sequence reads-tissues included data from the following projects: PRJNA510228, PRJNA435962, PRJEB21674, PRJEB8056, SAMN00715838, and PRJNA508405. A total 74 opium poppy tissue samples, collected from different ecotypes of opium poppy. These tissues are known to be involved in benzylisoquinoline alkaloid (BIA) biosynthesis and produce high levels of secondary metabolite content (e.g., stem and capsule). We selected high-quality RNA-Seq datasets based on read depth and coverage. We considered the availability of metadata (e.g., sample origin, experimental conditions) to ensure compatibility and relevance for co-expression analysis. We used Aspera (version 3.11.0) for faster download speed.

### 2.2 Data quality assessment and mapping

Raw sequencing reads were screened by using the FastQC tool (Version 0.11.9) [28] by running on the Linux operating system (Ubuntu 20.04 LTS). Low-quality reads and reads with primer and adapter sequences were trimmed by the fastp tool (version 0.20.1) [29] a high-performance read-processing tool. Reads with a quality score greater than 25 (Q > 25) were retained, with the last two base pairs of each read trimmed to minimize sequencing errors, which are typically higher at the 3′ end. Then, the clean reads of the opium poppy were mapped using Bowtie 2 (version 2.4.1) [30]. The PlantTFDB v5.0 database was applied to identify transcription factors (TFs) in the transcriptome data of opium poppy (Table S3). Genes involved in benzylisoquinoline alkaloids biosynthesis pathways, such as morphine, sanguinarine, noscapine, reticuline and papaverine, were identified using BLASTx tools against the NR and protein databases with a cut-off of E-value ≤ 1e^−5^, as well as via the KEGG pathway database and relevant literature review [2,[31], [32], [33]]. Benjamini-Hochberg multiple-testing correction was applied with an adjusted p-value (FDR) less than 0.05 considered significantly enriched for GO and KEGG enrichment analyses.

### 2.3 RNA-seq data normalization and gene expression analysis

To estimate gene expression levels among different samples, we used the Transcripts Per Million (TPM) method [34]. Based on the Bowtie results from individual samples on the poppy, the gene expression profile was calculated by the fast and bias-aware software Salmon tool (version 0.14.0) [35]. Salmon provides accurate expression estimates very quickly by using new algorithms (exactly, coupling the concept of quasi-mapping with a two-phase inference method). We also applied the Log2 transformation method to decrease residual variability. Genes that passed the |log2 fold change (FC)|≥ 1 and p-value ≤0.05 were chosen to construct the co-regulation network in opium poppy.

### 2.4 Construct Co-regulation networks

Batch effects and unwanted variation in RNA-Seq data can obscure biological signals. To correct for these, batch effects were removed using the ComBat function from the SVA package (v. 3.44.0) [36], which applies an empirical Bayesian approach [37]. Mean.only method was used to estimate and correct batch effects. A model matrix incorporating ecotype and other adjustment variables was constructed. Expression data were organized into a matrix and adjusted accordingly. To assess sample clustering, t-SNE (Rtsne v0.16) was used with perplexity = 5, max_iter = 100,000 parameters. Following batch correction, we constructed a co-expression network using the WGCNA package. This method groups genes with similar expression patterns into modules and associates them with biological traits. Outlier samples were removed before network construction. A soft-thresholding power (β = 5) was chosen using the *pickSoftThreshold* function, based on the scale-free topology criterion (fit index ≥0.8 and adequate mean connectivity). The step-by-step network was constructed using the following parameters: power = 5, corType = “bicor”, networkType = “signed hybrid”, TOMType = “signed”, minModuleSize = 30. The adjacency matrix was converted to a topological overlap matrix (TOM), and gene modules were identified through hierarchical clustering and the Dynamic Tree Cut algorithm. Closely related modules were merged using a module eigengene dissimilarity threshold of 0.20 [17].

### 2.5 Visualization of hub genes in network modules

Genes with a high correlation in modules involved in numerous interactions are considered hub genes, which play a more important role in a candidate module than other genes. In this study, the connectivity ranked in the top 10 % was set to identify hub genes as the most highly connected genes in a module based on the size of the module and KME. Genes with high module membership (kME >0.8) were representatives of the overall expression profile in the module, and genes with high module membership tend to be “hub” genes in the module (high intramodule connectivity) in this research. High KME values were measured to avoid the phenomenon of fuzzy clustering. The applied threshold ensured the selection of genes with strong module membership, emphasizing their functional significance and structural importance within the network. We aimed to obtain maximum similarity within each module and maximum difference between modules**.** The co-expression network was visualized using Cytoscape [38], and hub genes were detected in cytoHubba App (Version 0.1) based on Maximal Clique Centrality (MCC) and degree approaches [39].

### 2.6 GO enrichment screening and functional annotation

AgriGo web tool [40] was used for GO enrichment screening and functional annotation in opium poppy.

### 2.7 Linked protein network analysis

To construct the protein-protein interaction (PPI) network for hub genes, the STRING online tool (version 12.0) [41] was used. To assess potential interactions, the dicot genome annotation information was selected as the background. To determine whether co-expressed hub transcription factors (TFs) within each module remained significantly associated based on PPI information, PPI-linked network analysis was performed.

### 2.8 Promoter motif enrichment analysis in modules

We assessed motif enrichment analysis for genes in modules to understand particular cis-promoter motifs in each module. First, we determined 2000 bp upstream of the start translation codon as promoter regions. Motifs obtained from MEME [42]. This program was performed 50 times for each interested cluster and putative consensus motifs were detected based on letter-probability matrices and statistical modeling approaches. In order to detect putative TFs related to the motifs, significant motifs were evaluated by plantCARE [43], and New PLACE [44] databases in the promoter. Overrepresented promoter motifs of every gene in the module were identified.

### 2.9 Data validation: quantitative real-time PCR and ROC analysis

The expression levels of MYB as a regulatory TF and key genes NOS and CYP82, which are involved in noscapine biosynthesis, along with reference E.F.Iα in stem and capsule tissues of *P. somniferum.*These gene expression data were applied for partial least squares regression (PLS) analysis using sklearn.cross_decomposition in Python. The *corrplot* package (version 0.92) in R was used to visualize the correlation matrix and confidence intervals, with a confidence level of 0.95. Details regarding RNA extraction and qPCR analysis have been previously described in Refs. [45,46]. Receiver operating characteristic (ROC) analysis was performed to assess the potential of hub transcription factors (TFs) as co-regulatory genes within a specific metabolite pathway.

The *ggplot2* package was utilized to generate the receiver operating characteristic (ROC) curve, while the *pROC* package was used to compute the area under the ROC curve (AUC). AUC values served as a measure of predictive performance, with values above 0.7 indicating high predictive accuracy. Additionally, the partial AUC (pAUC) was derived from the ROC curve, which represents the relationship between the true positive rate (TPR) and the false positive rate (FPR) across different threshold levels. This analysis underscores the importance of ensuring that the classifier maintains a minimum level of sensitivity.

## 3 Results

### 3.1 Gene networks Co-expression and Co-regulation of the opium poppy transcriptome

**Fig. 1:**
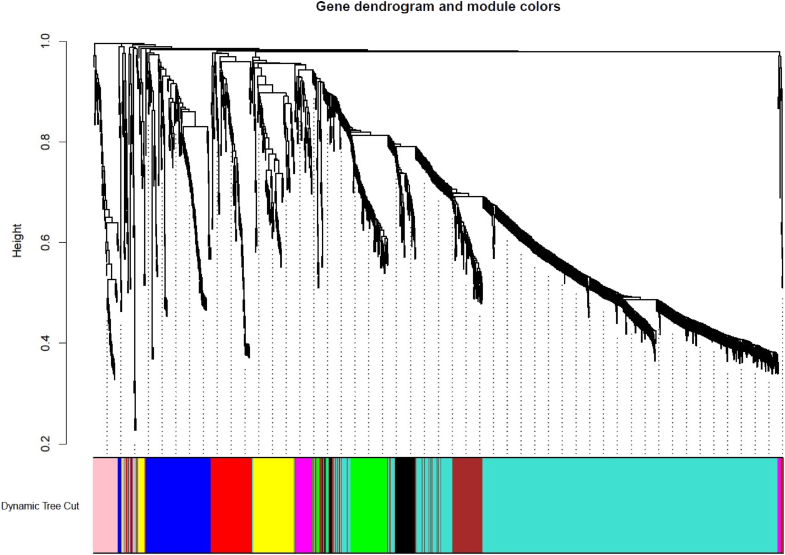
Hierarchical clustering of genes based on dissimilarity. The colored row below the dendrogram shows module membership assigned by the dynamic tree cut method, together with determined merged module colors.

To study the co-expression and co-regulatory network of genes in poppy, first, the raw RNA-seq data were filtered with FastQC and fastp tools, then high-quality reads of poppy samples were mapped by Bowtie2, and the TPM of genes was assessed by salmon. A total of 71584964 clean reads were obtained. 98.3 % of the raw sequencing reads were gained. Over 95 % of reads passed quality filters and were retained for downstream analysis. On average, approximately 92 % of the reads were successfully mapped, demonstrating high alignment quality appropriate for downstream transcriptomic analysis. After data preprocessing, we used expression matrices of 1501 genes and TFs involved in BIA, which were obtained from the 72 opium poppy samples, to construct a weighted co-expression network using the pairwise gene correlation method. Before performing network analysis, t-SNE was used on all transcriptome datasets to evaluate sample clustering patterns. As an unsupervised machine learning algorithm, t-SNE reduces dimensionality by grouping similar items based on proximity and distinguishing dissimilar ones by distance. We used this method to identify clustering patterns across different samples. These evaluation techniques enhanced accuracy and efficiency while minimizing errors. The results showed that homologous samples clustered together (Fig. S1). In most cases, the clusters of transcripts with similar expression patterns were of most interest for network analysis. Initially, normalized transcripts were hierarchically clustered based on the correlation values among them, serving as a distance measure. A dendrogram and correlation heat map were construed to represent topological overlap values between genes in the modules. Soft-thresholding power was chosen 5 for the scale-free topology model (Fig. S2). In total, nine modules were detected, with a maximum module size of 736 transcripts in turquoise and a minimum module size of 40 in magenta. The major arranged modules with different colors were turquoise (containing 736 genes and TFs), blue (145 genes and TFs), brown (113 genes and TFs), and yellow (101 genes and TFs). To identify relationships among modules, we conducted hierarchical clustering of module eigengenes and presented a heatmap plot illustrating the adjacencies in the eigengene network (Fig. S3). High module adjacency belongs to yellow and black modules whereas the pink and green modules had low adjacency. We investigated multiple dynamic merges by using a threshold value of 0.2 to detect correlation among modules (Fig. 3). This indicated no modules merged, and the greater number of modules eigengene pairwise correlations were more than 0.8 (Fig. 1. Table S4). High correlations were observed between yellow and black modules (R2 > 0.74). The correlation between the turquoise and brown modules was found to be R2 > 0.69. The eigengene correlations show the similarity in expression profiles among modules, which suggests possible co-regulation or shared biological functions. The notable BIA modules were related to turquoise, brown, and pink. The brown module comprises 49 genes and 64 TFs, and the pink module includes 24 genes and 64 TFs. In this study, most of the genes in the pink module (24 genes) are associated with benzylisoquinoline alkaloid (BIA) biosynthesis, particularly the codeinone pathway. The brown module includes 49 genes involved in noscapine biosynthesis. We conducted a bootstrap stability analysis (1000 iterations) to assess module robustness. The average gene-wise module stability score was 0.52, indicating that the majority of modules were consistently preserved across resampled datasets**.**

### 3.2 Identification of hub gene and visualization

**Fig. 2:**
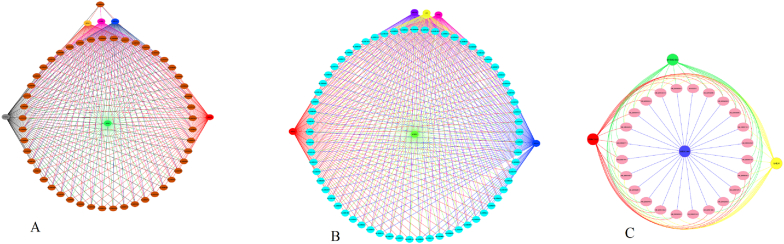
The co-regulation network analysis of hub TFs in opium poppy. Colored circles show genes, and edges show correlations among genes. Each TF in modules is shown with specific color. The remarkable BIA modules including brown module (A), turquoise module (B), and pink module (C) are revealed, respectively.

The most important genes in the scale-free topology network, known as hub genes, displayed a high degree of connectivity within a co-expression module. The hub TFs co-regulate several genes in the same pathway simultaneously. We identified several hub TFs in three notable modules associated with the benzylisoquinoline alkaloids pathway. The additional information on hub TFs genes in each module is shown in Table S2. Functional annotation and enrichment analysis of the hub genes and related genes were performed for each module (Table S5, Fig. 4). In total, 65 genes were found in the turquoise module, which were co-regulated by MYB3R-5, WRKY3, WRKY32, APRR2, and bZIP as hub TFs. WRKY48, WRKY12, MYB52, MYB43, and MYBAS2 were identified as hub TFs in the brown module, which are the indispensable factors in the benzylisoquinoline alkaloids pathway, especially participating in noscapine biosynthesis. Hub TFs participated in the pink module containing WRKY44, WRKY40, bHLH, and MYB82. Most of the genes in pink are related to the codeinone pathway. TFs highly co-regulated with genes involved in benzylisoquinoline alkaloids are shown in Fig. 2.

**Fig. 3:**
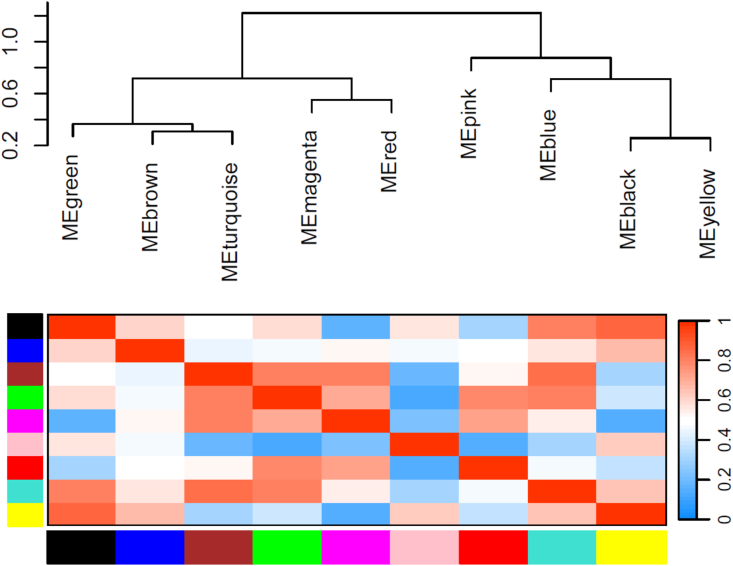
The details on hierarchical clustering dendrogram of MEs with dissimilarity according to topological overlap and relationship among modules of opium poppy. Each row and column in the heatmap relate to one module eigengene which is labeled by a specific color. Red color reveals high adjacency, while blue color reveals low adjacency in the heatmap.

**Fig. 4:**
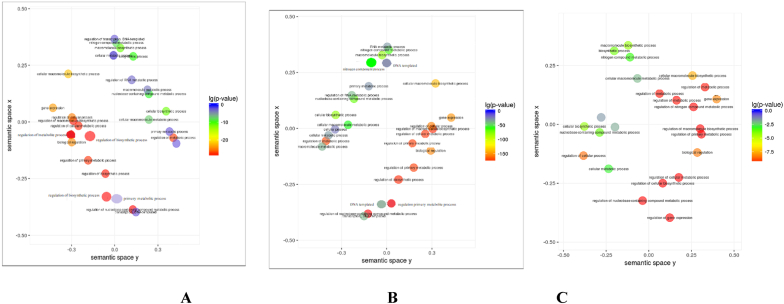
Functional enrichment analysis results of modules. (A) brown module, (B) turquoise module, (C) pink module. The size of the circles indicates the enriched number of genes, and the color of the circles indicates the significance level of the adjusted p-value.

**Fig. 5:**
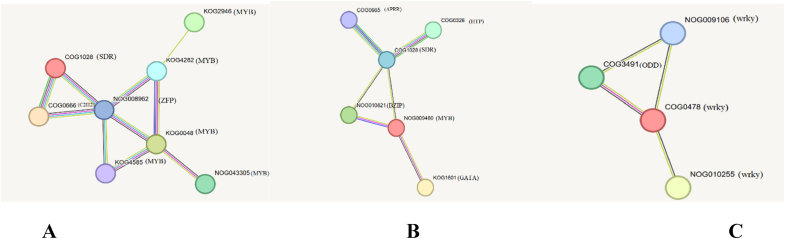
Protein-protein interactions network of hub TFs related to BIA modules including brown module (A), turquoise module (B), and pink module (C) in opium poppy based on string analysis are shown, respectively.

The PPI network outcomes revealed notable connections and strong associations among most hub genes due to their regulatory roles in cellular systems. The PPI networks based on hub TFs from three key modules related to benzylisoquinoline alkaloid biosynthesis showed strong connectivity, demonstrating the effectiveness of our approach in organizing functional modules of proteins with similar functions. These modules may impact the regulatory mechanism ofBIA secondary metabolite biosynthesis, supporting the validity of our results (Fig. 5).

### 3.3 Regulatory elements in promoter regions relate to genes within modules

Coordinated regulation of module genes may have similar regulatory motifs. TFs are one of the major components that bind to the specific origin of the transcription initiation site in the promoter. We conducted motif analysis of the 2 kb promoter regions of these opium poppy genes using the MEME program to determine the common motifs to understand which genes regulate each other. Then we found several TFBSs of many recognized TFs in the promoter regions of these gene modules by using the New PLACE and PlantCARE databases (Table S7). The enriched motifs in BIAs modules include CCAAT-box, MYB, and W-box. MYB and WRKY transcription factors were associated with AACCA and TGAC, respectively. These TFs play a key role in regulating secondary metabolites. The most significant *cis*-elements associated with TFs inducing MYB, WRKY, ZFP, and bZIP were predominant in the brown, pink, and turquoise modules, respectively. These hub genes, associated with BIA modules, highlight the essential roles of these factors. These TFs often serve as key positive regulators in plant metabolite biosynthesis pathways [47,48]. Based on transcription factor binding sites (TFBSs) screening, multiple unique TFBSs of hub TFs in the promoter regions of co-expressed genes in modules related to benzylisoquinoline alkaloids of opium poppy were discovered (Table 1). The results recommend that secondary metabolic genes be divided into the same module that may be regulated together by the same TF.

**Table 1:** Consensus motifs identified in the promoter region of genes of modules involved in secondary metabolite biosynthesis from opium poppy.

	Consensus motifs	Hub TF	Web logo
Brown module	ATCTGGTCAGTRRCA	WRKY	
	TAGYTCACTTTTTCT	MYB	
Pink module	AAMGAAAAGGAAGAA	MYB33	
	CSTGACACTGAGTGA	WRKY	
	AGGGGTGCAACTGCR	BHLH	
Turquoise module	GCGTCACCCGATTTA	ARR	
	GTTCWTCYTGGTTTG CCAACCAATTTCTGC TGGCTCGGCAACTC	MYB	


	TCGGTGAATCATTTG ACWCGTGCAGGKRAC	bZIP	

	CAGCATGACGAGCCC	WRKY	

### 3.4 Data validation

Partial Least Squares (PLS) is a method within artificial intelligence and machine learning. It is primarily used for modeling relationships between independent and dependent variables, especially in high-dimensional data situations where multicollinearity exists among predictors. PLS regression constructs latent variables that maximize covariance between these sets of variables, facilitating predictive modeling and dimensionality reduction [49]. We used PLS analysis to validate our results. PLS analysis was executed to reveal the influence of regulon hub genes on their targets in transcriptome sequencing data samples from stem and capsule tissues in opium poppy whose BIA contents were also evaluated [32]. The use of PLS statistical analysis has been employed to confirm the network results, enhancing the robustness of the findings. PLS analysis also revealed the effective association and strong effect of hub genes on the expression of controlled genes. PLS analysis showed a considerable effect of MYB26 and WRKY12 on the genes involved in noscapine biosynthesis, especially NOS in the brown module. Interestingly, the result of our analysis was nearly consistent with the WGCNA results which confirmed the role of the identified hub TFs in the regulation of BIA biosynthesis in opium poppy (Fig. S4). It seems that there is coordinated gene expression in this condition, so the transcription factor could increase the expression of those genes involved in benzylisoquinoline alkaloid biosynthesis. Our findings revealed that more effective and crucial hub TFs such as MYB 33, BHLH, ARR, WRKY 12, and bZIP could be important regulatory genes for regulating the benzylisoquinoline alkaloids biosynthesis pathways.

**Fig. 6:**
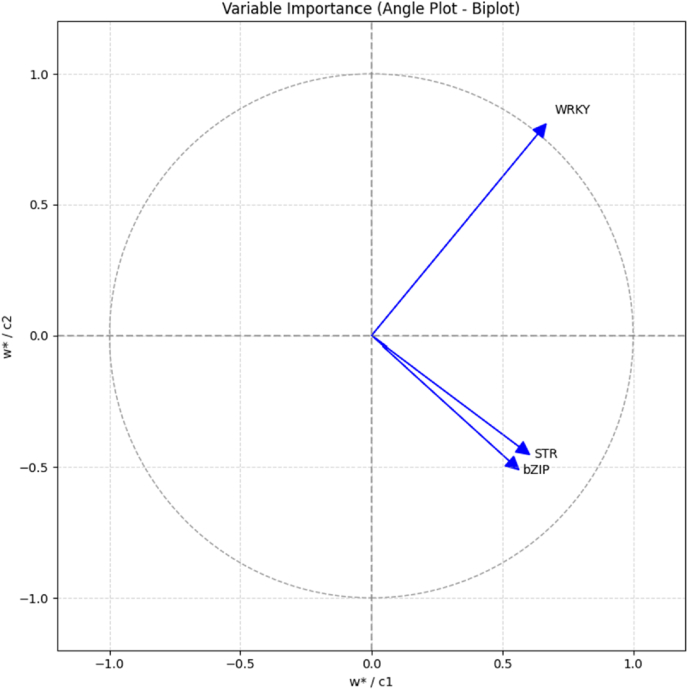
Partial Least Squares regression (PLS) was carried out to determine complex relationships between TFs and related genes in the turquoise module.

PLS statistical analysis has been employed to confirm the laboratory results, enhancing the robustness of the findings on qPCR data samples [45,46,50]. PLS analysis revealed a strong association and significant impact of hub genes on the expression of related genes. It also showed a considerable effect of WRKY 40 and bZIP on the genes involved in benzylisoquinoline alkaloid biosynthesis, especially STR (Fig. 6).

**Fig. 7:**
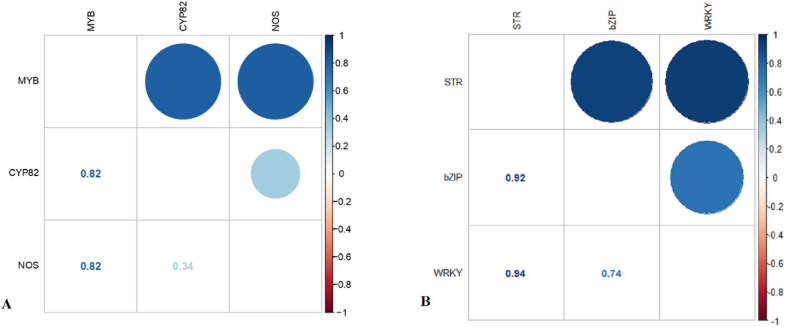
A graphical representation of a correlation matrix, confidence interval, and significant levels between hub TFs and genes in the brown and turquoise modules in opium poppy.

The results confirmed that hub TFs, especially MYB26 had a high correlation (P < 0.05) and a positive effect on genes under their control in benzylisoquinoline alkaloid biosynthesis (NOS, CYP81) in the brown module among stem and fruit tissues (Fig. 7 A). A strong correlation was found between the expression of hub TFs such as WRKY 40 and bZIP and the relevant gene STR in the turquoise module (Fig. 7 B). Our findings revealed that more effective and crucial hub TFs such as MYB 33, BHLH, ARR, WRKY 12, and bZIP could be important regulatory genes for regulating the benzylisoquinoline alkaloids biosynthesis pathways. Also, A strong correlation was observed between gene expression and BIAs in opium poppy. Specifically, a significant correlation was observed between the levels of morphine and the expression of WRKY hub TF in the stem. Notably, noscapine strongly correlated with MYB hub TF (R^2^ > 1) in fruit (Fig. S5).

**Fig. 8:**
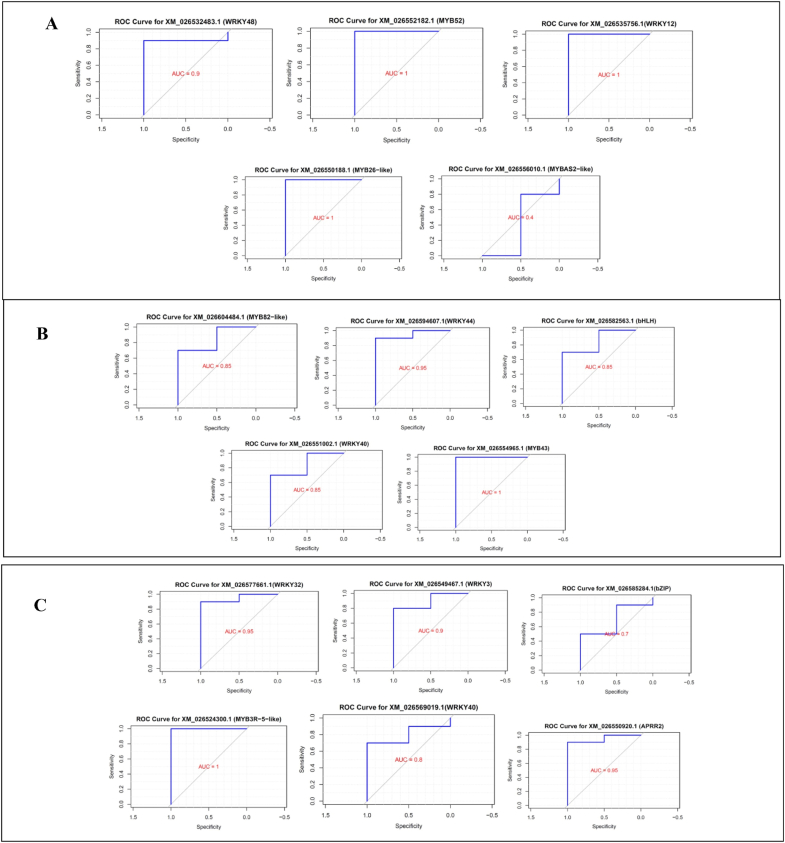
ROC analysis of hub TFs in significant co-regulation modules related to BIAs in opium poppy. The ROC curves assess the performance of these hub TFs involved in benzylisoquinoline alkaloid (BIA) biosynthesis. Panels A, B, and C represent the ROC curves and their corresponding hub TFs from the brown, pink and turquoise modules, respectively.

ROC analysis revealed that hub TFs such as MYB26 AUC: 1(brown module), MYB43 AUC: 1 (pink module), and MYB3R AUC: 1 (turquoise module) had high diagnostic value and could serve as co-regulatory factors for genes with the same module of BIAs in opium poppy. ROC analyses for each hub TFs are shown in Fig. 8. The pAUC provided a valuable metric for evaluating binary classifiers by emphasizing performance in the most relevant regions for regulatory considerations (Fig. S6). Overall, the selected hub TF from significant co-regulation modules, with higher ROC values, presents promising candidates as a regulatory factor involved in secondary metabolite biosynthesis in opium poppy.

Furthermore, we showed that these hub TFs control benzylisoquinoline alkaloid biosynthesis by interacting with the promoters of various benzylisoquinoline alkaloid biosynthetic genes by sharing common regulatory motifs.

## 4 Discussion

Co-expression networks and their module analysis define interactions among genes and reveal the common biological processes and molecular mechanisms among them. We created an opium poppy transcriptional network using a WGCNA method and identified hub genes related to secondary metabolites using transcriptome data from different ecotypes. Correlation analysis among co-expression modules was performed, and 9 modules were recognized; three of these modules had the most important genes related to benzylisoquinoline alkaloids, namely turquoise, brown, and pink. Usually, genes within the same module have the same transcriptional regulation. Combinatorial protein interactions of transcription factors may affect the exact regulation of module genes [51]. Modules frequently describe groups of transcripts that share regulations or pathways [52]. Modules with a high level of eigengenes correlation display a tight relation in transcript content, which recommends that gene regulation may be similar. This suggests that they affect alkaloid productivity. The results also indicated that the same transcription factor can regulate multiple genes, and one module can synchronously be related to various transcription factors. Hub genes of the module are usually illustrated as representative of a given module in the biological network and can play control roles in the expression of related genes. Our findings validate that regulatory hub TF genes are involved in coordinating the expression of genes associated with secondary metabolite biosynthesis. Transcription factors such as MYB and WRKY can play important roles in essential regulatory machinery for BIA biosynthesis [53,54].WRKY TFs are known as regulators of secondary metabolism in plants. Several studies have determined the regulatory role of WRKY in the secondary metabolism of the plant. WRKY proteins can directly bind to W-box elements in promoters of genes involved in alkaloid biosynthesis, modulating their expression. For example, a WRKY was revealed to activate berberine biosynthetic genes, enhancing alkaloid accumulation in *Coptis japonica*. WRKY are also implicated in stress-responsive regulation, which often correlates with secondary metabolite production. This suggests that WRKYs may integrate environmental signals to regulate alkaloid biosynthesis pathways [[54], [55], [56]]. MYB TFs are a large family known for their roles in regulating secondary metabolites, such as the alkaloid pathway. In the opium poppy, MYB TFs have been identified that regulate key enzymes in morphine biosynthesis. MYB proteins typically bind to MYB recognition elements in target gene promoters, activating or repressing transcription. It is proposed that overexpression of hub TFs, such as WRKY, using tissue-specific promoters might result in more BIAs. Some studies have shown that overexpression of these TFs in opium poppy, *Catharanthus* spp and *Taxus media* could promote increases in flux through the alkaloids pathway by regulating and activating expressions of a series of genes, confirming their regulatory roles [53,[57], [58], [59]]. The existence of two to four W-boxes in the promoter region of several genes involved in artemisinin biosynthesis in *Artemisia annua* was identified by Yang et al. (2015) [60]. This study also recommends that AaWRKY is capable of promoting the expression of these genes [60]. Additionally, overexpression of AaWRKY1 caused a rise in artemisinin accumulation (1.8-fold) [61] in *Artemisia annua* plants was reported. In this study, the transcription factors MYB family, WRKY family, bHLH, and bZIP were known as hub TFs in BIAs modules. In addition, genes involved in noscapine biosynthesis pathways were enriched in the brown module. Salutaridine reductase (SalR), the crucial enzyme related to morphine biosynthesis, was found in the turquoise module. Also, the pink module was enriched in codeinone.

The roles of hub TFs such as WRKY and MYB have been determined and confirmed in previous research in opium poppy by qPCR. For instance, the study conducted by Rezaie et al., 2017 [62] highlighted the role of WRKY, whose expression pattern corresponds to BIA accumulation in opium poppy fruits. The results demonstrated a strong coordination and positive correlation between the expression of WRKY transcription factors and the genes involved in the biosynthesis of BIAs. Notably, the highest level of coordination was observed between the expression of the WRKY transcription factor and the TYDC gene. Since the TYDC gene is among the initial genes involved in BIA biosynthesis, this transcription factor likely exerts influence over the entire biosynthetic pathway by modulating the expression of this gene. MYB genes in opium poppy have further confirmed their regulated role in BIA metabolism. The qRT-PCR analyses show the presence of these TFs in all organs of opium poppy. Specifically, these TFs were revealed to regulate BIA biosynthesis by interacting with the promoters of genes involved in the secondary metabolite pathway [45]. Studies showed high gene expression levels of TFs and related secondary metabolite genes were observed in the main tissues producing secondary metabolites [11,12,22,46]. The studies confirmed that TFs such as MYB, WRKY, and bZIP can control the genes involved in benzylisoquinoline alkaloid biosynthesis [45,50,62]. Our findings discovered that more effective and crucial hub TFs, such as MYB 33, BHLH, ARR, WRKY 12, and bZIP could be important regulatory genes for regulating the BIA biosynthesis pathways. Our correlation analysis between gene expression of hub TFs and secondary metabolite concentration data revealed a strong association between the gene expression and metabolome in opium poppy*.* Previous studies have reported a strong relationship between the transcriptome and metabolome in various plant species, including *Artemisia annua* L., *Allium sativum*, *Allium*. *umbilicatum*, *Allium*. *fistolosum*, *Allium*. atroviolaseum, *Allium*. *rubellum*, and *Allium*. *stamineum*, *Dracocephalum kotschyi* L., *Trigonella foenum-graecum L.*, *Crocus sativus* L., and opium poppy [11,12,46,60,[63], [64], [65], [66], [67]]. Many Recent studies were exhibited on metabolome and Transcriptome levels to reveal the regulation mechanisms in plants [[68], [69], [70], [71]]. The regulation of secondary metabolites is strongly regulated at various levels, particularly at the transcriptional level [72]. Recent studies confirm the significant role of RNA in the regulation of secondary metabolite production among plants [68,73].

TFs with high connectivity within BIA modules showed significant associations in the PPI network, suggesting that Hub genes were also linked at the protein-protein interaction level.

The Promoter is the main region of transcription where transcriptional elements are assembled and control gene expression. Therefore, one of the powerful approaches to understanding the co-regulatory mechanism of genes is to investigate the promoter regions of genes. Promoter structure can describe the regulatory properties of genes. The properties of coregulated genes depend on their promoter structure [74]. The presence of common *cis*-acting elements among genes of the co-expression modules can be determined by performing promoter analysis. Consensus promoter motifs of modules involved in secondary metabolite production were determined in this study. MYB and WRKY TFs commonly control various genes associated with the metabolite biosynthesis pathway by utilizing identical *cis*-acting elements present in their promoters [45,[75], [76], [77]]. Additionally, promoter motifs associated with WRKY and MYB transcription factors were frequently identified in the promoters of genes involved in noscapine biosynthesis in opium poppy [45]. The promoter analysis confirmed the presence of binding sites for key transcription factors, which may further explain how genes within modules are co-regulated. The presence of identical *cis*-regulatory elements in the promoter regions of genes within the same module suggests shared regulation, implying that these genes may function within a common biological pathway [78]. Such elements serve as molecular signatures of co-regulated gene modules, reflecting control by common transcription factors and enabling coordinated expression within a regulatory network. Previous studies indicated that overexpression of PsWRKY as an important hub gene enhanced alkaloid production. PsWRKY activated the promoter of the TYDC gene in yeast [53,79].

Genomic clustering verifies that the co-expression of BIA genes increases within clusters [32]. Previous studies have identified gene clusters related to noscapine and thebaine pathways [33,80]. Genes situated close together on chromosomes are expected to be co-regulated and share *cis*-regulatory elements. Genomic clustering plays a significant role in the co-expression of BIA genes in opium poppy. Studies have shown that these genes, which are essential for the biosynthesis of important alkaloids like noscapine and thebaine, tend to be organized into clusters on chromosomes. This spatial arrangement facilitates coordinated expression and regulation, which is crucial for metabolic productivity [32,80,81]. We observed co-expression and co-regulation among the modules related to clustering genes in the brown and turquoise modules, which enriched the genes of the noscapine and thebaine pathways, such as CYP81, NOS, and SalAT, respectively. These clusters not only improve co-expression but also influence regulatory mechanisms. The RT-qPCR analysis showed high gene expression levels of hub TFs, and related benzylisoquinoline alkaloid genes were observed in the main tissues producing secondary metabolites [45,46]. PLS and ROC analyses confirmed the potential role of hub transcription factors (TFs) involved in BIA pathways. PLS and WCGNA approaches strengthen confidence in WRKY and MYB TFs as key regulators by combining unsupervised network discovery with supervised feature association. Moreover, WGCNA can guide the identification of candidate TFs for further validation, while PLS ranks those with the strongest regulated effects, allowing a more detailed understanding of MYB and WRKY-mediated regulation of BIA biosynthesis. Co-regulation network analysis and the exploration and isolation of common regulatory hub TFs could provide new insight for breeding quantitative traits, such as promoting the production of secondary metabolites. This goal can be achieved by engineering specific regulatory genes, which are usually more efficient than complex step [82].

## 5 Conclusion

The opium poppy is a primary source of essential medicinal benzylisoquinoline alkaloid metabolites. In this study, we analyzed its transcriptome data to construct a co-regulatory network. Nine modules related to opium poppy secondary metabolites were recognized. Three specialized modules were enriched and had the maximum number of genes involved in the benzylisoquinoline alkaloid biosynthesis pathway. Hub TFs, such as MYB26 and WRKY, which played a regulatory role in BIAs biosynthesis, and consensus motifs in the module related to these hubs were identified. These hub TFs can be utilized to enhance the production of these pharmaceutically important compounds by simultaneously upregulating multiple genes and expression systems in platforms such as cell suspension cultures and hairy root systems for regulon engineering. PLS and ROC analysis identified the possible and functional role of hub TF involved in the BIA pathways. To reveal the role of genes involved in BIA biosynthesis, it has been suggested to use gene knockout techniques such as CRISPR-Cas9 and RNA interference (RNAi) techniques. The ChIP-seq technique is suggested for future regulatory motifs relevance studies. We have determined that the results obtained hold significant value, particularly at the onset of research concerning co-regulatory network analysis in medicinal plants, notably in the opium poppy. The co-regulatory network analysis of opium poppy offers new insights into the specific regulatory mechanisms of benzylisoquinoline alkaloid biosynthesis at the transcriptome level and paves the way for regulon engineering of candidate hub TFs in expression systems platforms.

## Author contributions

ME: Investigation, Formal analysis, Software, Visualization, Writing – original draft, Methodology. SRM: Conceptualization, Methodology, Data curation, Writing - review & editing, Visualization. All authors have read and approved the final manuscript.

## Consent for publication

Not applicable.

## Data availability

The datasets generated during and/or analyzed during the current study are available in the National Center for Biotechnology Information (NCBI) repository, https://www.ncbi.nlm.nih.gov/sra. The details of the accession number of SRA data are shown in Table S1.

## Funding

This work was also supported by 10.13039/501100008257Tarbiat Modares University (Grand number: 89852).

## Declaration of competing interest

The authors declare that they have no known competing financial interests or personal relationships that could have appeared to influence the work reported in this paper.

## Abbreviations

**TF**: Transcription Factor
**WGCNA**: Weighted Correlation Network Analysis
**t**-**SNE**: t-distributed stochastic neighbor embedding
**TOM**: Topological Overlap Matrix
**KEGG**: Kyoto Encyclopedia of Genes and Genomes
**NCBI**: National Center for Biotechnology Information
**PLS**: Partial Least Squares regression
**qRT-PCR**: Quantitative real-time PCR
**ROC:**: Receiver Operating Characteristic
**AUC**: area under the ROC curve
