# Supplementary material for: Network-based identification of hub transcription factors associated with benzylisoquinoline alkaloid biosynthesis in Papaver somniferum
Source: Biochem Biophys Rep. 2025 Jul 23;43:102147. doi: 10.1016/j.bbrep.2025.102147 (PMC12307667; doi:10.1016/j.bbrep.2025.102147)
Supplement: Multimedia component 1 [file mmc1.docx]

**Supplementary Material**

**Network-Based Identification of Hub Genes Associated with Benzylisoquinoline Alkaloid Biosynthesis in *Papaver somniferum***

**Mahsa Eshaghi^1^, Sajad Rashidi-Monfared***^1^

^1^Department of Plant Biotechnology, Faculty of Agriculture, Tarbiat Modares University, Tehran, Iran.

^1^Department of Plant Biotechnology, Faculty of Agriculture, Tarbiat Modares University, Tehran, Iran.

* Corresponding author.

E-mail address: rashidims@modares.ac.ir (S. Rashidi-Monfared)

**Figure. S1**. T-SNE analysis to disclose the arrangement of different ecotypes of opium poppy according to the transcriptome sequencing data.

**Figure. S2**. Soft threshold assignment process used to achieve the scale-free topology index

**Figure S3.** Side view of Topological Overlap heatmap plot in the gene network. Light and dark colors represent low and high topological overlap respectively. also, the module assignment and gene dendrogram are showing the top the left side.


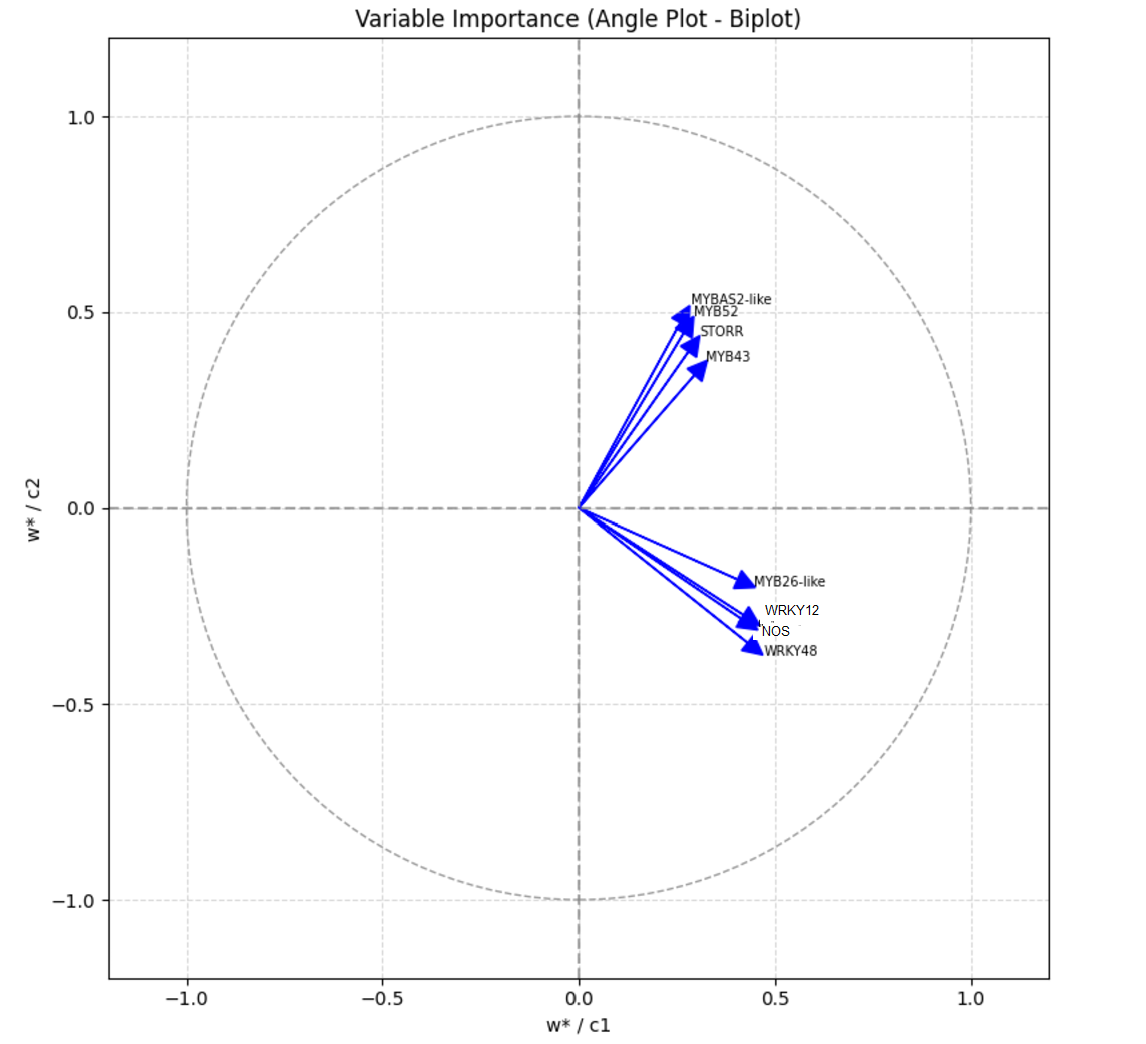

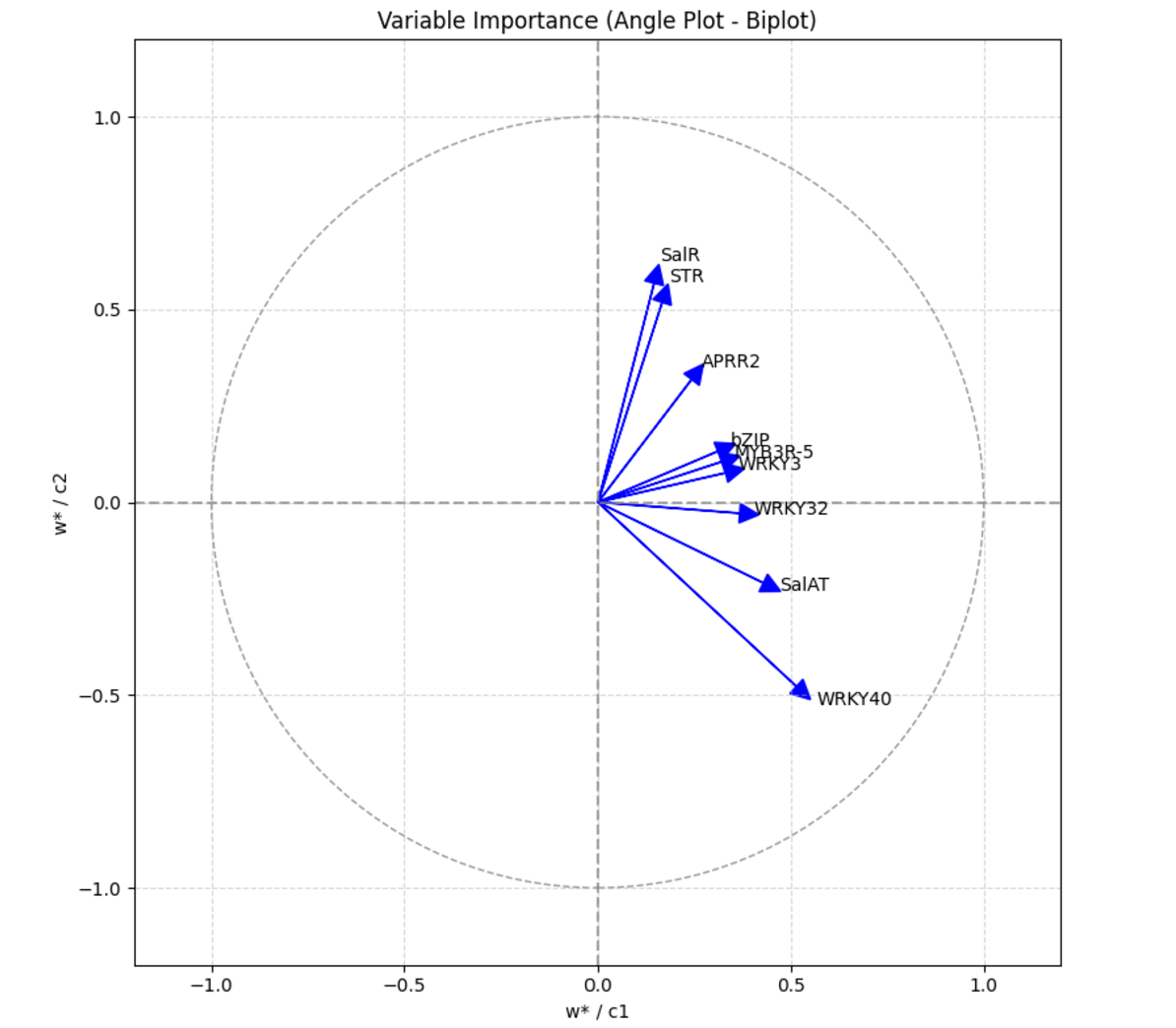

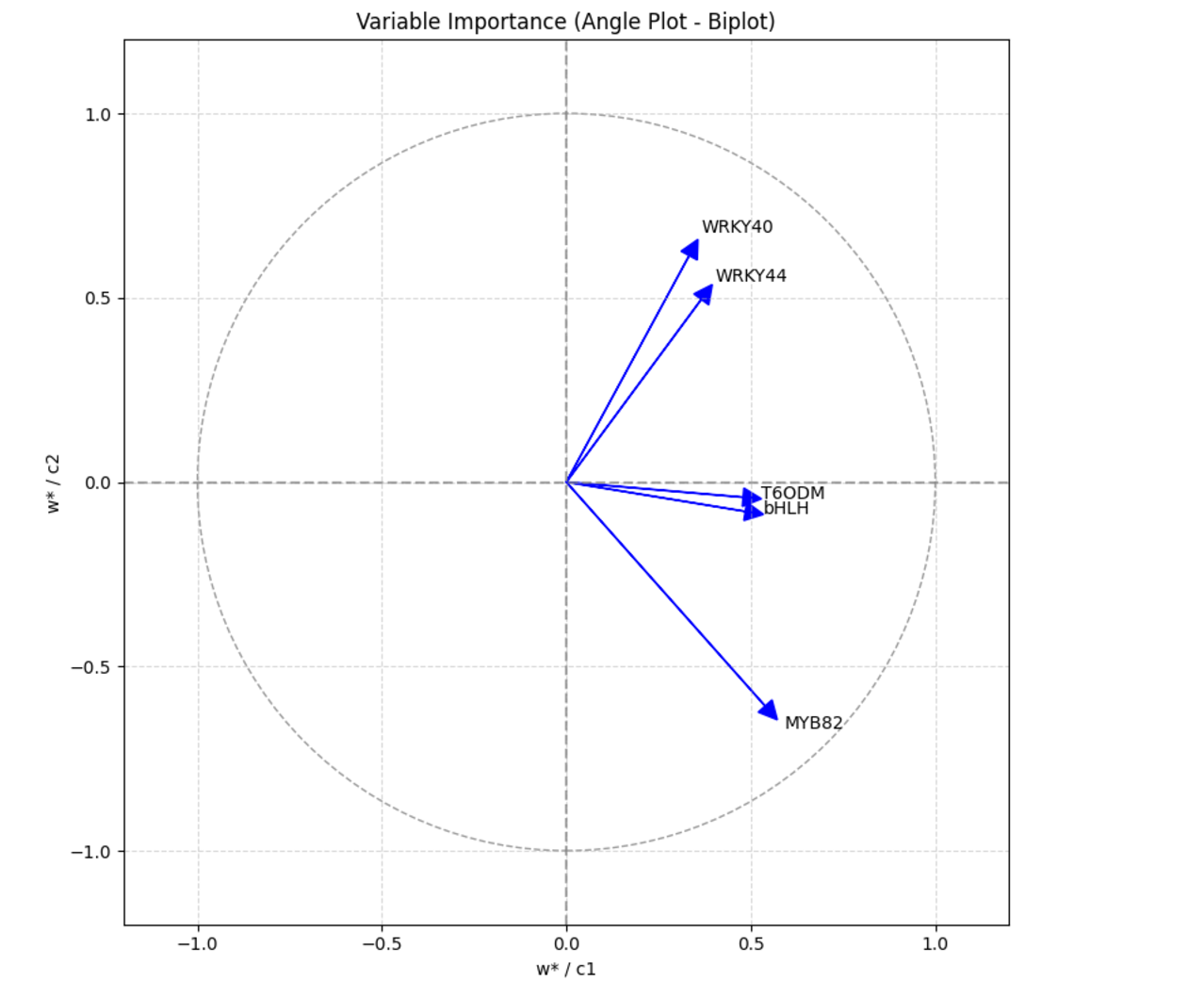


**A B C**

**Figure S4.** Partial Least Squares regression (PLS) was carried out to determine complex relationships between TFs and related genes in important modules. Brown module (A), turquoise module (B), and pink module (C).

**Figure S5.** Correlation analysis between gene expression of the hub genes and metabolite concentration (Rezaei et al., 2017). The confidence level was considered 0.95.

**Figure S6.** pAUC analysis of hub TFs within important modules. The green rectangle shows the sensitivity and the blue rectangle shows specificity. Panels A, B, and C represent the pAUC curves and their corresponding hub TFs from the brown, pink, and, turquoise modules, respectively.

**C**


**B**

**A**
